# Evaluation of the Effects of Sativex (THC BDS: CBD BDS) on Inhibition of Spasticity in a Chronic Relapsing Experimental Allergic Autoimmune Encephalomyelitis: A Model of Multiple Sclerosis

**DOI:** 10.5402/2012/802649

**Published:** 2012-08-07

**Authors:** A. Hilliard, C. Stott, S. Wright, G. Guy, G. Pryce, S. Al-Izki, C. Bolton, G. Giovannoni

**Affiliations:** ^1^GW Pharmaceutical PLc, Porton Down Science Park, Wiltshire SP4 0JQ, UK; ^2^Barts and The London School of Medicine and Dentistry, London E1 2AT, UK

## Abstract

This study investigated the antispasticity potential of Sativex in mice. Chronic relapsing experimental allergic encephalomyelitis was induced in adult ABH mice resulting in hind limb spasticity development. Vehicle, Sativex, and baclofen (as a positive control) were injected intravenously and the “stiffness” of limbs assessed by the resistance force against hind limb flexion. Vehicle alone caused no significant change in spasticity. Baclofen (5 mg/kg) induced approximately a 40% peak reduction in spasticity. Sativex dose dependently reduced spasticity; 5 mg/kg THC + 5 mg/kg CBD induced approximately a 20% peak reduction; 10 mg/kg THC + 10 mg/kg CBD produced approximately a 40% peak reduction in spasticity. Sativex has the potential to reduce spasticity in an experimental mouse model of multiple sclerosis (MS). Baclofen reduced spasticity and served as a positive control. Sativex (10 mg/kg) was just as effective as baclofen, providing supportive evidence for Sativex use in the treatment of spasticity in MS.

## 1. Introduction

Spasticity may be best understood as an increase in muscle tone leading to muscle hypertonia and exaggerated tendon reflexes [1]. It is a painful symptom that develops during multiple sclerosis (MS) and spinal cord injury, where damage to the nervous system results in uncontrolled limb motor function [2]. Like most other symptoms of MS, spasticity is caused as a result of demyelination and nerve loss within neural circuitry [3]. There are several types of spasms that affect people with MS. They tend to be asymmetrical and, due to slow or interrupted nerve impulses, muscles either do not relax as quickly as they should, tighten involuntarily, or stay contracted for longer periods of time [2]. Spasticity is a significant problem for around 60% of MS patients [1], and it has been estimated that one-third of people with MS alter their daily activities due to spasticity [3].

Current forms of treatment for people suffering from MS-related spasticity include physical therapy, which involves stretching and hydrotherapy, employment of mechanical aids, such as braces, use of chemical blocks including phenol injected into the muscles or intrathecally, or in extreme cases, surgery [4, 5]. The most common form of treatment is the use of oral medications, but current therapy is often associated with dose-limiting adverse side-effects. Approximately 37% of MS patients take the most commonly prescribed antispasticity agents [2] including baclofen, dantrolene, tizanidine, diazepam, gabapentin, botulinum toxin, and phenol, but many clinicians report that a proportion of these patients are not being adequately treated due to adverse effects of these medications.

For the same reason, some patients have turned to self-medication and have perceived benefit from cannabis [6]. Sativex (nabiximols) is derived from extracts of the *Cannabis sativa Linnaeus *plant, with a 1 : 1 mixture of delta-9-tetrahydrocannabinol (THC) and cannabidiol (CBD) as the main active substances, delivered as an oromucosal spray. The principal pharmacological effects of THC include analgesic, muscle relaxant, antiemetic, appetite stimulant, and psychoactive effects [7]. CBD has anticonvulsant, muscle relaxant, anxiolytic, neuroprotective, antioxidant, and antipsychotic activity [8], though its importance lies not only in its own inherent therapeutic profile but also in its ability to modulate some of the undesirable effects of THC through both pharmacokinetic and pharmacodynamic mechanisms [9]. 

Sativex has recently been licensed for the treatment of spasticity in MS following a number of positive clinical trials [10]. A meta-analysis of 666 patients with MS who had spasticity that was not adequately controlled using existing treatments and were then given Sativex (*n* = 363) or placebo (*n* = 303) showed that there was a definite reduction in patient reported problems, that the effects of Sativex were usually evident within three weeks, and that about one-third of people given Sativex as an add-on treatment gained at least a 30% improvement from baseline [11]. Similarly, a double-blind, randomized trial of Sativex in 337 subjects with symptoms of spasticity due to MS demonstrated that Sativex treatment resulted in a significant reduction in treatment-resistant spasticity [12]. 

The cannabinoids within Sativex target the cannabinoid receptors, designated CB_1_ and CB_2_, which are concentrated in the central nervous system (CNS) and peripheral terminals of primary afferent neurons and the immune system [13]. The cannabinoid system consists of a number of endogenous fatty acid ligands together with biosynthetic and degradation systems and has been shown to regulate synaptic neurotransmission [14, 15]. Therefore, it may be anticipated that Sativex would control spasticity, which is a product of uncontrolled nervous signaling. We investigated this by assessing the effect of the botanical drug substances (BDSs), which comprise Sativex in spasticity in chronic relapsing experimental autoimmune encephalomyelitis (CREAE), an animal model of MS [16]. A cannabis extract containing 20% THC but no CBD has previously been shown to treat experimental spasticity in EAE at a dose of 5 mg/kg i.v. [17]. However, a 1 : 1 mix of CBD : THC was chosen for this study as this is the medicinal product currently licensed and used for the treatment of spasticity in patients with MS, with all the associated benefits that the combination of the two cannabinoids provide to patients in a clinical setting.

## 2. Materials and Methods

### 2.1. Animals

Adult (20–25 g; 6–8 weeks old) Biozzi ABH mice were bred at Queen Mary University of London or purchased from Harlan-Olac UK, (Bicester, UK). Animals received a subcutaneous injection of 1 mg freeze-dried mouse spinal cord homogenate in Freund's adjuvant supplemented with 60 *μ*g *Mycobacterium tuberculosis *and *Mycobacterium butryicum* (8 : 1) in the flank on Days 0 and 7 as described previously [16]. This induced the development of a relapsing-remitting paralytic disease with the accumulation of an increasing neurological deficit. Assessment of tremor and spasticity involves the use of animals in remission, usually after the second or third relapse 40–80 days after inoculation [18]. In this study, mice were used 7-8 months after inoculation. Again they were in postrelapse remission and exhibited paresis and visual evidence of spasticity [18] but by this stage, were very severely affected. The mice with residual paresis and visible evidence of spasticity during postrelapsing progressive EAE were then randomized into four parallel groups (*n* = 6–8 per group) that were treated with either: 1 : 1 THC : CBD at a dose of 5 mg/kg, 1 : 1 THC : CBD at a dose of 10 mg/kg, baclofen at a dose of 5 mg/kg as a positive control, or vehicle alone.

### 2.2. Compounds

BDSs containing high levels of either THC (69.3%) or CBD (66.5%) were supplied by GW Pharma Ltd. and held under a Home Office Licence to Possess under The Misuse of Drugs Act 1971. These were combined in a 1 : 1 ratio based on the amount of principal cannabinoid within each BDS, then dissolved in ethanol prior to addition of Cremophor (Sigma, Poole Dorset) and then phosphate-buffered saline (PBS) in a ratio of (1 : 1 : 18). This closely resembles the composition of Sativex, where the same BDSs are formulated in a 50 : 50 mixture of ethanol and propylene glycol, and for simplicity is referred to as Sativex throughout this paper. Baclofen was purchased from RBI/Sigma (Poole, UK) and was dissolved in PBS. All compounds were injected intravenously in the tail vein using a 30 g needle in a volume of 0.1 mL at doses of 5 mg/kg or 10 mg/kg Sativex and 5 mg/kg baclofen. The doses of Sativex used were chosen on the basis of previous studies [18, 19] and represent a reasonable equivalence to the clinical dose range used in human patients. 

### 2.3. Assessment of Effects

The “stiffness” of spastic limbs was assessed by measuring the force required to bend the hind-limb of each mouse to full flexion using a purpose-built strain gauge. This evaluation has been used effectively in numerous previous studies [17, 18, 20–22] and is the only reported tool for investigating spasticity related to MS in animals. Moreover, it provides objective and qualitative readouts. Its application in this study was further validated by the use of a positive control. Both the left and right hind-limbs were assessed repeatedly, typically five times per time point, with the resulting analogue signals being amplified and then digitized and captured using a DAQcard 1200 PCMICA card (National Instruments Austin, TX, USA) and Acquire V1 software (D. Buckwell, Insititute of Neurology, University College London) on the Windows XP platform. The data were analysed using Spike 2 software (Cambridge Electronic Design, UK), and the forces were converted to Newtons (N). Limbs showing severe crossing or flexion were not analysed and limbs exhibiting flexion forces of less than 0.15 N, (i.e., within the range of limb stiffness for normal animals [18]) were not analysed resulting in some animals or some limbs being excluded from the final data. Taking this into account, each group contained a minimum of five animals. A mean score for each limb at each time point was calculated as well as a group mean score based on all available hind-limb data for all available mice within each group. The results represent the mean ± SEM resistance to flexion force (N), which were compared using repeated measures analysis of variance (RM ANOVA) incorporating Student-Newman-Keuls post hoc test. Statistics were performed using Sigmastat V3/Sigmpalot V9 software (Systat Software Inc, Hounslow UK).

## 3. Results

**Figure 1:**
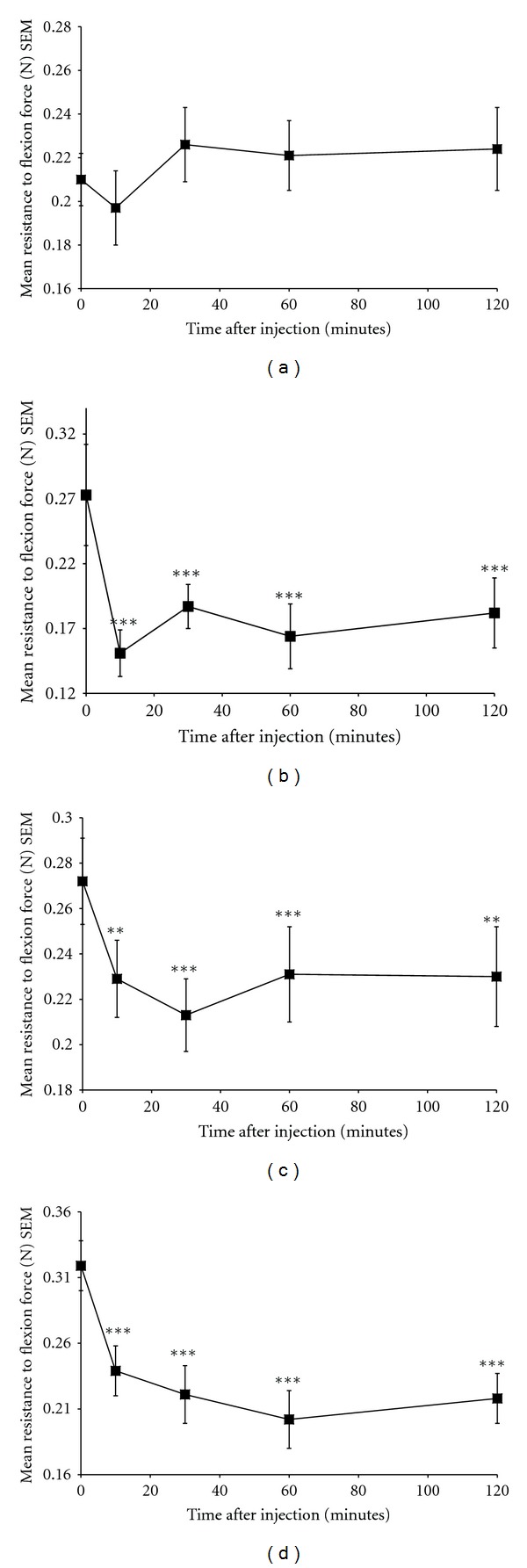
Mean (SEM) change in resistance to flexion force following treatment with (a) Vehicle, (b) 5 mg/kg Baclofen, (c) 5 mg/kg Sativex and (d) 10 mg/kg Sativex.

**Figure 2:**
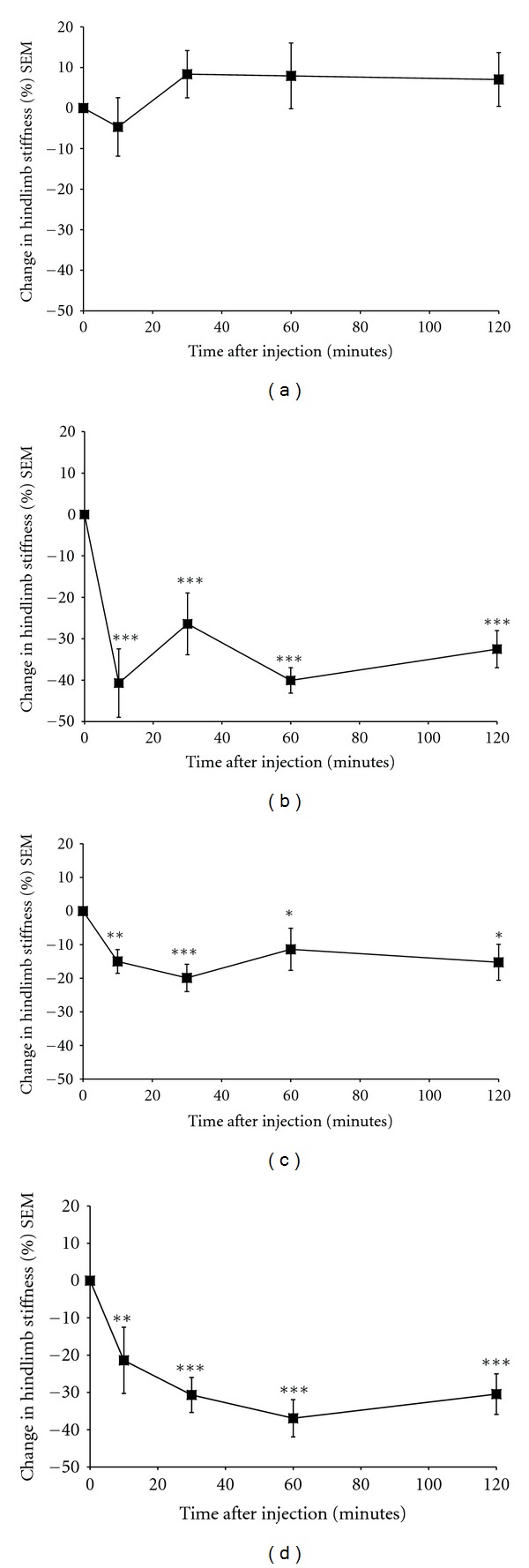
Mean (SEM) percentage change from baseline in hindlimb stiffness following treatment with (a) Vehicle, (b) 5 mg/kg Baclofen, (c) 5 mg/kg Sativex and (d) 10 mg/kg Sativex.

Vehicle injection failed to induce a significant (*P* < 0.3) change in the degree of spasticity over a two-hour observation period (mean [SEM] percentage change from baseline after 120 minutes = 7.05 [6.63]) (Figures 1(a) and 2(a)). Baclofen served as a positive control and after 5 mg/kg i.v. injection into spastic mice, induced a significant reduction in the degree of limb stiffness compared to baseline. This was evident within 10 minutes of treatment (mean [SEM] percentage change from baseline after 10 minutes = −40.71 [8.26]; *P* < 0.001) and persisted for the two-hour observation period (mean [SEM] percentage change from baseline after 120 minutes = −32.51 [4.48]; *P* < 0.001) (Figures 1(b) and 2(b)). This treatment-induced marked limpness and hypomotility, as assessed visually in the recipient mice, is consistent with the mechanism of action of GABA_B_ receptor agonists.

Treatment with the formulated Sativex BDSs at a dose of 5 mg/kg THC + 5 mg/kg CBD i.v. induced a significant inhibition in the degree of limb stiffness. This was evident within 10 minutes of treatment (mean [SEM] percentage change from baseline after 10 minutes = −14.99 [3.54]; *P* = 0.007) and an inhibitory effect could be detected two hours after administration (mean [SEM] percentage change from baseline after 120 minutes = −15.25 [5.9]; *P* = 0.011) (Figures 1(c) and 2(c)). This treatment induced some mild sedation, as assessed visually, in the recipient mice which is consistent with the mechanism of action of CB_1_ receptor agonists.

Treatment with the formulated Sativex BDSs at a dose of 10 mg/kg THC + 10 mg/kg CBD i.v. induced a significant inhibition in the degree of limb stiffness. This was evident within 10 minutes of treatment (mean [SEM] percentage change from baseline after 10 minutes = −21.40 [8.87]; *P* = 0.002) and maintained for at least two hours (mean [SEM] percentage change from baseline after 120 minutes = −30.44 [5.45]; *P* ≤ 0.001) after administration (Figures 1(d) and 2(d)). This level of inhibition of spasticity was comparable to that seen previously with 5 mg/kg i.v. baclofen (Figures 1(b) and 2(b)). This treatment dose induced some sedation as assessed visually in the recipient mice, and is again consistent with the mechanism of action of CB_1_ receptor agonists.

In summary, vehicle treatment had no effect on spasticity (Figures 1(a) and 2(a)). Sativex BDSs administered at a dose of 5 mg/kg THC + 5 mg/kg CBD i.v. produced an approximate 20% peak reduction in hind limb stiffness (Figures 1(c) and 2(c)), whereas Sativex BDSs administered at a dose of 10 mg/kg THC + 10 mg/kg CBD i.v. produced an approximate 40% peak reduction in hindlimb stiffness (Figures 1(d) and 2(d)). This was highly comparable with baclofen administered at a dose of 5 mg/kg i.v., which also produced an approximate 40% peak reduction in hindlimb stiffness (Figures 1(b) and 2(b)). It, therefore, took a higher dose of Sativex compared with baclofen to achieve the same reduction in spasticity; however on a mg per mg basis, Sativex BDSs were observed to be better tolerated than baclofen as baclofen-treated mice were much more immobile than the Sativex BDS-treated animals. This was, however, a very subjective finding based on observation alone, and mouse movement in the cage was not actually quantified.

It should also be noted that for mice receiving baclofen or either of the Sativex doses, the resulting reduction in spasticity was still very much in effect two hours after dose. Given the chronic nature of spasticity due to MS and the need of patients for ongoing treatment, any future studies could provide invaluable information about the duration of action of Sativex compared with baclofen by extending the length of time that animals were monitored over.

## 4. Discussion

Cannabis has long been proposed for its effects as an antispasmodic and muscle relaxant [23]. This study provides evidence that the cannabinoid compounds within cannabis used to produce Sativex have the potential to dose dependently inhibit spasticity in an experimental mouse model of MS. This study also provides evidence that the antispasticity effect of Sativex treatment in this model are comparable to the effects produced by the current most commonly used form of oral antispasticity treatment, baclofen. The vehicle proved to be inactive, causing no reduction from baseline in the level of spasticity in this model. Although baseline values for flexion forces were initially higher in the vehicle group, it should be noted that mice were not randomized on preassessed spasticity readings which is why initial group means were sometimes different as individual limbs can vary dramatically from limb to limb and animal to animal [18]. Furthermore, based on previously published data, the baseline values, although varied, do not influence whether a positive drug effect can be identified [18]. 

Side-effects of baclofen are well known and result in limitations in its clinical application and use [24]. It is used clinically at doses from 15 mg/day up to 80 mg/day, with doses as high as 200 mg/day being used safely; such a dose represents about 3 mg/kg/day. Given the rapid metabolism in rodents, this equates to about a 10-fold difference, such that the dose we examined in rodents would be within the realms of human use. However, even though the dose which has been used previously in animals [25] demonstrated that baclofen has antispastic capability, it also presented adverse behavioral effects. In comparison, the dose of Sativex required to provide the same antispasticity effect resulted in fewer side effects with mice showing only mild sedation. Although the difference in observable side effects was quite marked in this study, leading to the assumption that they would also be clinically different, it would have been helpful to have quantified the differences seen and to have gathered information on how the differences would translate in terms of clinical benefit through further qualitative behavioral testing. 

Importantly, following abrupt withdrawal of baclofen in humans, a number of significant adverse behavioural effects including visual and auditory hallucinations, convulsions (status epilepticus), dyskinesia, confusion, psychotic, manic or paranoid states, anxiety with tachycardia, and sweating, and insomnia, to name a few, have been observed [26–28]. Withdrawal from baclofen treatment, therefore, requires a slow dose reduction, over a period of several weeks, and there are limitations with respect to the population groups in which baclofen can be used. In particular, its use in elderly patients and patients with cerebrovascular disorders or a history of psychiatric illness is limited due to its associated withdrawal symptoms. Unlike baclofen, studies into long-term use of Sativex in patients with MS have shown that sudden discontinuation of treatment does not result in any significant withdrawal-like symptoms [29, 30], although some people did report temporary changes in their sleeping patterns, emotional status, and appetite following discontinuation. This suggests that Sativex could be used as a safe and effective treatment alternative, since the lack of withdrawal symptoms suggest that dependence on this treatment is highly unlikely.

The duration of bioavailability of baclofen within the CNS is known to have the ability to constantly activate all GABA_B_ receptors in the brain, causing prolonged GABA_B_-mediated responses. This could explain the well-documented dose-related side effect profile of this compound and highlights that such first-line therapies may not be able to reach their therapeutic potential due to lack of tolerability. As such, the need for more effective treatments still remains. Sativex works independently of GABA_B_ receptor mechanisms and the most common side effects reported by people taking Sativex are dizziness and fatigue, thought to be CB_1_ receptor-mediated effects. These common side effects of Sativex in human studies have been shown to occur in the first four weeks of treatment and become less frequent over time [31]. In addition, unlike all other antispasticity medication, Sativex is a self-titrating oromucosal medicine, allowing each user to achieve their maximum tolerated dose alongside symptom relief. For patients who do develop side effects, the option to reduce future doses or increase the time between doses enables them to reduce the likelihood of future recurrence.

Previous clinical studies have indicated that THC administered at doses between 5 and 10 mg, given orally, significantly relieved spasticity compared with placebo and displayed a large treatment effect [32, 33]. Even though these small studies reported minimal adverse effects, the belief that the majority of Sativex-related side effects are CB_1_ receptor mediated, alongside the well-known psychotropic properties of THC via CB_1_ receptor activity, means that THC alone remains very limited in its clinical application. Synthetic derivatives of THC have been produced. One clinically available compound is nabilone, which has been investigated and shown to relieve muscle spasms better than placebo in the clinic; however, this study only observed a single case [34]. As with THC, nabilone binds with high affinity to the CB_1_ receptor, but acts as a full agonist at CB_1_ whereas THC is a partial agonist. This inevitably increases the likelihood of adverse side effects with nabilone compared to THC, again resulting in limitations in its clinical use. 

These and other studies performed over time have indicated the potential of THC as an antispasticity agent; however, more recent availability of standardised cannabis medicinal extracts for preclinical and clinical research have enabled the understanding of the therapeutic potential of other cannabinoids, aside from THC. It has been suggested that CBD, which is the major nonpsychoactive cannabinoid component of cannabis, may modify the pharmacokinetics and pharmacodynamics of THC to antagonise some of the undesirable effects caused by THC at the CB_1_ receptor [9, 35]. It has also been proposed that CBD may have awakening properties and may work as both a CB_1_ and CB_2_ receptor antagonist [13], which in turn could counteract the potential cannabimimetic effects of THC. The mild sedation observed with Sativex in the ABH mice at both doses, an effect consistent with THC agonism via CB_1_ receptors, may well be reduced through its CBD content. Overall, the development of Sativex, and research into the potential synergistic effects between CBD and THC coadministration may extend the clinical application and therapeutic benefits of THC alone.

The developed tolerance of Sativex in patients may be supported by the internal regulation of the endocannabinoid system. Endocannabinoids are produced *de novo* in response to physiological stimuli [36], and levels of endocannabinoids appear to be dysregulated in various pathologies including MS and spasticity [18]. Studies have shown that in CREAE mice with spasticity, levels of endocannabinoids are increased compared with normal and nonspastic CREAE mice, providing definitive evidence of the underlying involvement of the endocannabinoid system in the regulation of spasticity mechanisms [20]. The endocannabinoid system plays a role in the homeostasis of various endogenous functions [37], and Sativex could potentially affect the underlying involvement of the endogenous cannabinoid system in the regulation of the manifestations of spasticity. Further investigations into the changes in endocannabinoid levels in response to Sativex may lead to a greater understanding of its mechanistic effect. More recently, CBD alone has been suggested to be an inhibitor of the primary hydrolysing enzyme of the endocannabinoids [38], fatty acid amide hydrolase, in turn increasing the levels of endogenous CB_1_ receptor agonists, such as anandamide. This could cause further agonism of the CB_1_ receptor located in presynaptic neurons, resulting in the inhibition of neurotransmitter release, thus contributing to the reduction in spasticity observed [39, 40].

 There is good evidence that THC is a partial CB_1_ agonist and that the CB_1_ receptor is key in the pathogenesis of spasticity. In the absence of CB_1_ receptors, cannabinoid receptor agonists do not elicit antispastic effects [22]. Furthermore, the use of CB_1_ receptor antagonists has been shown to increase spasticity in this animal model. Abnormalities of the endocannabinoid system have recently been shown in MS in humans [41]. This provides mechanistic and pharmacological evidence that the cannabinoid constituents found in Sativex are therapeutically beneficial in the treatment and control of spasticity in MS, primarily via this receptor. Another element to the CREAE model is the development of additional MS-related symptoms including tremor and spasticity [18]. The beneficial effects of these compounds observed in this model provide a reasonable correlation to the treatment of spasticity as assessed in the clinic. Previous studies looking at the effect of Sativex in placebo-controlled randomized clinical studies show it to improve the symptoms of spasticity in MS sufferers [23]. 

## 5. Conclusions

The positive results from this study of Sativex in the CREAE model of spasticity in MS, together with data obtained from clinical studies in humans, provide increasing evidence for the beneficial use of Sativex in humans. Sativex at a dose of 10 mg/kg in this model is seen to be as effective and more importantly, elsewhere, has shown to be better tolerated than the first-line treatment currently used for MS-related spasticity, baclofen. Hence, we would regard Sativex to be a potential new contender for the first-line treatment of spasticity in patients with MS.
